# Assessing the epidemiological impact on cervical cancer of switching from 4-valent to 9-valent HPV vaccine within a gender-neutral vaccination programme in Switzerland

**DOI:** 10.1186/s12889-020-08840-0

**Published:** 2020-05-12

**Authors:** André B. Kind, Andrew Pavelyev, Smita Kothari, Nadia El Mouaddin, Aurélie Schmidt, Edith Morais, Patrik Guggisberg, Florian Lienert

**Affiliations:** 1 Department of Gynaecology and Gynaecological Oncology, University Hospital Basel, Spitalstrasse 21, CH-4056 Basel, Switzerland; 2 Center for Observational and Real-World Evidence (CORE), Merck & Co., Inc, Kenilworth, NJ 07033 USA; 3 HCL America, Inc., Sunnyvale, CA USA; 4 ICON plc, 55 avenue des Champs Pierreux, 92000 Nanterre, France; 5 ICON plc, 27 rue de la Villette, 69003 Lyon, France; 6 Outcomes Research, MSD, 162 Avenue Jean Jaurès, 69007 Lyon, France; 7 Market Access, MSD, Werftestrasse 4, CH-6005 Luzern, Switzerland; 8 Medical Affairs, MSD, Werftestrasse 4, CH-6005 Luzern, Switzerland

**Keywords:** HPV vaccination, Cervical disease, Impact, Gender-neutral vaccination, Switzerland, HPV, Cervical cancer, Vaccination, 9-valent HPV, Epidemiology

## Abstract

**Background:**

An infection with high-risk human papillomavirus (HPV) is the obligatory aetiological factor for the development of cervical cancer. In Switzerland, the prevention strategy for cervical cancer is based on primary prevention via HPV vaccination and secondary prevention with an opportunistic screening programme for precancerous lesions. Vaccination is recommended to 11–26 years old male and female persons. The objective of the study was to assess the epidemiological impact on cervical cancer of switching from the currently implemented programme with the 4-valent vaccine to the 9-valent vaccine, in an 11–26 years old gender-neutral vaccination programme in Switzerland.

**Methods:**

A previously validated dynamic transmission model of HPV infections was adapted and calibrated to the Swiss setting assuming an 80% coverage rate in HPV-vaccination and lifelong vaccine type-specific protection. A gender-neutral vaccination programme (males and females) for 11–26 years old with a 9-valent HPV vaccine was compared with the current 11–26 years old gender-neutral 4-valent vaccination programme. Sensitivity analyses were conducted in order to test the impact of lower vaccination coverage rates and a shorter duration of protection on the model outcomes.

**Results:**

In Switzerland, a 9-valent gender-neutral vaccination programme would result in an additional prevention of 2979 cervical cancer cases, 13,862 CIN3 and 15,000 CIN2 cases, compared with the 4-valent gender-neutral vaccination programme over 100 years. These additional disease cases avoided would correspond to a 24, 36 and 48% cumulative incidence decrease in cervical cancer, CIN3 and CIN2 cases, respectively. It would also prevent additional 741 cervical cancer-related deaths over 100 years. A substantial additional reduction in cervical cancer and precancerous lesions burden is still observed when varying the vaccination coverage rate from 30 to 60% or reducing the duration of protection from lifelong to 20 years.

**Conclusions:**

The switch to the 9-valent vaccine in Switzerland to prevent cervical diseases showed an important contribution in terms of public health impact compared with the 4-valent vaccine in an 11–26 years old gender-neutral population, even with very conservative assumptions such as low coverage rates or low duration of protection and limiting analysis to only cervical disease.

## Background

Human papillomavirus (HPV) is the most common sexually transmitted infection. Although most HPV infections are transient and clear up without intervention within a few months after acquisition, persistent HPV infections may cause pre-cancerous lesions and ultimately cancer. More than 200 HPV genotypes have been identified. At least 12 off these are classified as oncogenic High-Risk types (types 16/18/31/33/35/39/45/51/52/56/58/59), which are causal agents of HPV-related cancers including cervical, vaginal, vulvar, penile, anal and head and neck cancer [1, 2]. On the other hand, low-risk HPV types such as HPV 6 and HPV 11 cause 90% of genital warts as well as the rare but debilitating recurrent respiratory papillomatosis (RRP) [3, 4]. In Europe, HPV is associated with almost 100% of cervical cancer cases and causes around 87% of anal, 70% of vaginal, 29% of penile, 16% of vulvar and 7% of head and neck cancers in females and males [4].

The annual number of new disease cases related to HPV in Europe were estimated to be close to 35,000 cases of cervical cancer, 1500 cases of vulvar cancer, 1500 cases of vaginal cancer, 6500 cases of anal cancer, 1200 cases of penile cancer and 7000 cases of head and neck cancer [4].

In Switzerland, around 5000 new cases of cervical precancerous lesions (CIN2 and CIN3) and 250 new cases of cervical cancer are being diagnosed and result in an estimated 75 deaths each year. Cervical cancer is the fifth most frequent type of cancer among women aged 20 to 49 years [5, 6].

HPV 16/18 are the predominant HPV types in cervical diseases: 23–25% of CIN1 lesions, 38.4–39% of CIN2 lesions, 58% of CIN3 lesions and 73% of cervical cancer cases are caused by these two HPV types. The HPV 31/33/45/52/58 attributable fraction among HPV positive cases is estimated to be around 23–26% in CIN1 lesions, 32–35.9% in CIN2 lesions, 27–32% in CIN3 lesions and 16.2% in cervical cancer cases [7]. These international data on HPV attributable fraction have been confirmed in the Swiss setting. A recent study aiming to identify the HPV types present in the cervix tissue was conducted among 768 Swiss women diagnosed with CIN3+ between 2014 and 2015. The analysis showed that 61.8% of CIN3+ cases were associated with HPV 16/18, with an attributable fraction of HPV 31/33/45/52/58 of 27.7% (89.5% of cases were associated with an HPV genotype included in the 9-valent vaccine) [8, 9].

In Switzerland, the prevention strategy for cervical cancer is based on gender-neutral HPV vaccination (primary prevention) and screening (secondary prevention).

Cervical cancer screening in Switzerland is not provided through a national organized programme but on an opportunistic basis, meaning that cervical cancer screening is left to the initiative of women and doctors. However, cervical cancer screening based on cytology is recommended every 3 years for women aged 21 to 30 years and screening based on cytology or on primary HPV testing is recommended every 3 years for women aged 31–70 years [10]. Cervical cancer screening based on cytology for women aged 21 to 70 is reimbursed by Swiss health insurances.

In addition to screening, preventive gender-neutral HPV vaccination exists. Currently, there are three HPV vaccines approved in Switzerland. Two vaccines, targeting HPV 6/11/16/18 and HPV 16/18 were first licensed for clinical use in 2006 and 2010, respectively (Gardasil®, MSD, 4-valent HPV vaccine and Cervarix®, GSK SPC, 2-valent HPV vaccine) [11, 12]. Although both 4-valent and 2-valent HPV vaccines are recommended and approved in Switzerland, the 4-valent vaccine leads the HPV vaccines market with almost 95% of HPV vaccinations done with this vaccine (based on IMS data of MAT June 2017) [13]. This is mainly due to the fact that the 4-valent vaccine is the only HPV vaccine approved for the prevention of HPV-related diseases in boys and young men in Switzerland [11, 12]. A third vaccine (Gardasil 9®, MSD), was approved by the Swiss regulatory body Swissmedic in July 2016 for protection against nine HPV types (6, 11, 16, 18, 31, 33, 45, 52 and 58) and expected to provide increased coverage against the majority of high-risk HPV types with carcinogenic properties [14].

HPV vaccination was first recommended in Switzerland to girls and young women in 2007, and was then extended to boys and young men in 2015 by the Swiss Federal Office of Public Health (BAG) and the Federal Vaccination Commission (EKIF). The BAG and EKIF stated the following reasons for switching to a gender-neutral vaccination programme: a significant burden of HPV-associated diseases including genital warts occurring in men, protection of specific risk groups (men having sex with men - MSM), good safety and efficacy profile of HPV vaccination in males, and improved herd protection for both male and female. With the introduction of gender-neutral vaccination, men were no longer excluded from the benefits of the HPV vaccination [15]. It was also argued that gender-neutral HPV vaccination allows both sexes to bear responsibility for issues around sexual and reproductive health [15]. Recently other countries in Europe have also expanded their programme to a gender-neutral vaccination (e.g. Germany, Ireland and UK) [16–19]. The Swiss HPV vaccination programme is based on a two-dose regimen for girls and boys aged 11–14 years and a three-dose regimen for persons aged 15 to 26 years. Vaccination uptake and coverage rate in Switzerland was estimated around 69 and 54% respectively among 18–20 year old and close to 42 and 34% among 21–24 year old [20]. Due to the fact that the HPV vaccination programme is implemented on the cantonal level, a substantial variability in the HPV vaccination coverage rate in girls aged 16 is observed among the 26 Cantons, ranging from 30% in the Canton of Obwalden to 79% in the Canton of Valais [21].

The epidemiological impact of HPV vaccination on incidence and prevalence of HPV infections and HPV-related diseases has been extensively studied following the change of vaccination programme from a 4-valent to a 9-valent vaccine in many countries. Several European mathematical models have estimated the potential impact of switching from a 4-valent to a 9-valent HPV vaccination on the burden of HPV-related diseases or infections [22–24]. These studies, conducted in Austria, Germany and Italy, provided similar conclusions on the positive impact of the switch to a gender-neutral 9-valent vaccination on HPV-related diseases such as cervical cancer and pre-cancerous lesions [22–24].

In Switzerland, no such analysis on the epidemiological benefits of a 9-valent vaccination compared with the current vaccination programme has been done so far. Thus, the objective of this analysis was to estimate the epidemiological impact of switching to a 9-valent HPV vaccination programme for 11–26 years old boys and girls from the currently implemented programme with the 4-valent vaccination in Switzerland. This analysis considered the currently available 4-valent vaccine as the relevant comparator since almost 95% of current HPV vaccination is performed with this vaccine in Switzerland [13].

## Methods

### Mathematical model

A previously published and validated transmission model, simulating the natural history of genotypes 6/11/16/18 HPV-infections, has been extended to account for infections and diseases attributable to HPV genotypes 31/33/45/52/58. A detailed description of the model can be found in Boiron et al. [21] and Elbasha et al. [24–26] In summary, the dynamic model structure accounts for herd protection effects and herd immunity.).

An epidemiological module simulates HPV types 6, 11, 16 and 18 separately, whereas the five additional types (31, 33, 45, 52 and 58) are combined into a single set of compartments. The model accounts for the transmission dynamics of those nine HPV types and simulates the occurrence of genital warts; RRP; pre-cancerous lesions (CIN1–3); cervical, vulvar, vaginal, penile, anal, and head and neck cancers related to these HPV types.

For the Swiss adaptation of the model, only the following HPV-related diseases were included in the analysis: cervical cancer, CIN1, 2 and 3.

Despite that HPV causes vaginal, vulvar, anal, head and neck, and penile cancers, as well as RRP and genital warts, these diseases were not included in the analysis and will not be presented in the following sections for three main reasons. (i) The estimated incidence of anal cancers attributed to the HPV types 31, 33, 45, 52, 58 is relatively low. The overall incidence of HPV-associated anal cancers and HPV-associated head and neck cancers in Switzerland is estimated to be 155 cases and 73 cases, respectively [4, 15]. 2.9% of HPV-associated anal cancers and 3.7% of HPV-associated oropharyngeal cancers can be attributed to the 5 HPV additional types in the 9-valent vaccine [4]. This means that less than 4 additional anal cancer cases and less than 8 additional oropharyngeal cancer cases could be prevented every year with the 9-valent vaccine compared with the 4-valent vaccine. (ii) While there is no data available on the incidence of RRP, vaginal, vulvar and penile cancer in Switzerland, the estimated number of cases attributed to the HPV types 31, 33, 45, 52, 58 is low (less than 7 cases per year). (iii) 90% of genital warts are caused by HPV types 6 and 11. As both the 4-valent and 9-valent vaccine cover these types, no difference between prevented cases is expected.

### Model inputs

The model inputs, including data on demographics, sexual behaviour, screening, natural history of the disease, treatment patterns, cancer mortality and vaccination coverage were adapted to the Swiss setting.

### Demographics

Population data was issued from the Federal Office of Statistics (BFS). The total population in Switzerland at the end of 2015 was estimated to be 8,327,126 people [25].

### Sexual behaviour

Sexual behavioural data in Switzerland is scarce and therefore the results from the UK-NATSAL III (the third National Survey of Sexual Attitudes and Lifestyles) survey were used [26]. According to expert opinion (AK) this data is applicable to Switzerland. The extent of sexual mixing (value between 0 and 1 with 0 representing no mixing and 1 representing maximum mixing) among members of different age cohorts and sexual activity groups was extracted from the technical model report accompanying the publication by Elbasha et al. “Impact of Vaccinating Boys and Men against HPV in the United States” and adjusted during the calibration process [27].

### Screening

The percentage of females receiving gynaecological pre-cancer screening tests at least once every 3 years (75%) was derived from a study by Boiron et al. [22] and the proportion of women who received a follow-up screening test after an abnormal PAP result was set to 95% [28]. Age-specific coverage in the past year were extracted from a study by Burton-Jeangros et al. [29] and validated by expert opinion. In terms of diagnostic performance, sensitivity and specificity values of the screening tests were derived from Elbasha et al. [30].

### Natural history of the disease

The progression from infection to disease follows a similar history structure as the initial US model [30]. As transmission rates are not directly observable, the US values were used and adjusted through calibration techniques to obtain the set of parameters that best fit the Swiss data on cervical cancer and associated mortality.

### Disease and treatment patterns

The female population receiving hysterectomy over the course of 1 year was estimated from inpatient operation statistics for 2015 published by the Federal Office of Statistics/ Bundesamt für Statistik (BFS). The age-specific incidence of hysterectomies was calculated as the total number of hysterectomies by age divided by the sum of women by age group in Switzerland as reported by the BFS [25].

### Mortality

The annual probability of death for each HPV-related cancer, stratified by age and stage, were derived from the Austrian survival age-specific data from the EUROCARE-5 database [31, 32].

### Vaccination strategy

Annual HPV 4-valent vaccination coverage data from Switzerland in 2016 were used to inform vaccination coverage rates in the model [21]. It was assumed that 68% of girls aged 11–14 years were vaccinated in the first year. This percentage was based on the 56% of 16-year-old girls having received two doses of the 4-valent HPV vaccine in 2016 and assumes that the vaccination coverage rate (VCR) at launch of the 9-valent vaccine would be higher than the observed rate for the 4-valent vaccine in 2016. For boys aged 11–14 years, the VCR was assumed to be around 20% in the first year of the 9-valent vaccine introduction. A catch-up cohort of girls and boys aged 15–26 who had not been previously vaccinated or had not undergone a complete vaccine schedule was also considered assuming a VCR at launch of the 9-valent vaccine of 45% in women and 10% in men. VCR for both females and males aged 11–26 years was assumed to increase up to 80% in 10 years’ time.

As vaccines are assumed to have a similar clinical effect in all countries, vaccine properties parameters were kept the same as in the core model and were based on clinical trial data (Table 1) [33–38]. The duration of protection against HPV genotypes included in the 9-valent vaccine was assumed to be lifelong in the base case scenario. The efficacy for one dose was assumed to be 0%, efficacy for two doses was assumed to be 100% and herd immunity was considered. Compliance for second dose was assumed to be 95%. For people aged 15–26 an additional third dose was considered in the model as recommended by the Swiss vaccination schedule. Efficacy and compliance for this third dose was assumed at 100%.

**Table 1:** Summary table on vaccine efficacy assumptions for HPV-related cervical cancer

Vaccine assumptions	HPV 16	HPV 18	HPV 31/33/45/52/58
Vaccine efficacy for preventing cervical HPV16/18/31/33/45/52/58 infections:			
Male^a^	0.411	0.621	0.411
Female^b^	0.76	0.963	0.76
Degree of protection of the vaccine against cervical HPV16/18 infections becoming persistent	0.988	0.984	0.988
Degree of protection of the vaccine against HPV16/18-related CIN	0.979	1	0.979

^a^Preventing male genital infections through male vaccination is assumed to prevent transmission of genital infections to females

^b^Preventing female genital infections through vaccination is assumed to prevent transmission of genital infections to males

Source: Giuliano et al. (2011) [33] for males and Elbasha and Dasbach [30] for females

### Model calibration and validation

The calibration process aims to tune epidemiological inputs in order to obtain results closer to observed data. The targets of the calibration are the incidence and mortality rates of HPV-related diseases. Swiss HPV-related diseases overall incidence and mortality data were collected from the National Institute for Cancer Epidemiology and Registration (NICER) [39, 40] and the Federal Office of Public Health (BAG) [41] websites. Since the model studies HPV-induced cancers only, target epidemiological values were calculated by multiplying the incidence or the mortality with the percentage of the disease that can be attributed to HPV infection. The proportions of diseases attributable to HPV infection were collected from two publications by Hartwig et al. [42, 43].

The calibration process involved many rounds of iterations to move model outcomes closer to the targets. The following model outcomes were compared against the calibration target in each iteration:

- Cervical cancer incidence
- Mortality rate of cervical cancer

Several model variables (including behavioural parameters, natural history of disease, transmission rates, and all-cause mortality) were first adjusted by changing transmission rates. Then the specific parameters (e.g. rate of mortality and rate of seeking treatment for most cancers) were used to fine-tune each disease area.

### Epidemiological impact analysis

Two strategies were compared in this study:

- 9-valent vaccination for girls and boys aged 11–26 years in addition to the current cervical screening programme
- 4-valent vaccination for girls and boys aged 11–26 years in addition to the current cervical screening programme

The epidemiological impact of HPV vaccination strategies was calculated by estimating the total number of disease events prevented related to the HPV types 16/18/31/33/45/52/58; the reduction in HPV 16/18/31/33/45/52/58 infection prevalence; the incidence and mortality reduction of cervical cancer and the incidence reduction of CIN1–3. The cumulative percentage reduction of 9-valent vaccination versus 4-valent vaccination was calculated by estimating the incidence of each HPV-related disease for each year of the simulation for each scenario, and then by calculating the difference of actual number of cases of each HPV-related disease between the two scenarios on the population level and over the entire time period (usually 25, 50 and 100 years). Results were reported at 25 years, 50 years and 100 years for the different strategies tested.

Sensitivity analyses were performed deterministically, modifying the value of one base case parameter at a time. The two key parameters tested in this analysis are the maximum vaccination coverage rate (60% in males and females, 45% in males and females, 45% in females and 30% in males, 30% in males and females) and the duration of protection (20 years).

## Results

### Calibration

The model accurately replicated the published cervical cancer incidence and mortality rates (less than 1% difference). Indeed, the model reported the same values than observed cervical cancer incidence and mortality in females for the 4-valent vaccine (4.25/100,000 cervical cancer cases and 1.18/100,000 cervical cancer deaths) and similar values for the 9-valent vaccine (5.47/100,000 cervical cancer cases predicted in the model versus 5.46/100,000 cases observed, and 1.51/100,000 cervical cancer deaths reported in the model versus 1.52/100,000 cervical cancer deaths observed).

### Epidemiological/health outcomes

**Fig. 1:**
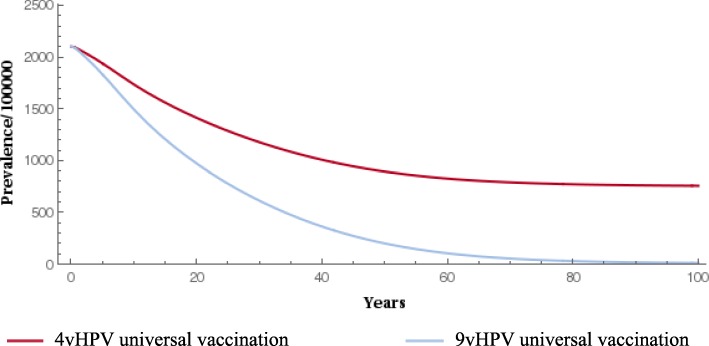
Epidemiological impact of two vaccination strategies on HPV16/18/31/33/45/52/58 infection prevalence among females over 100 years

**Fig. 2:**
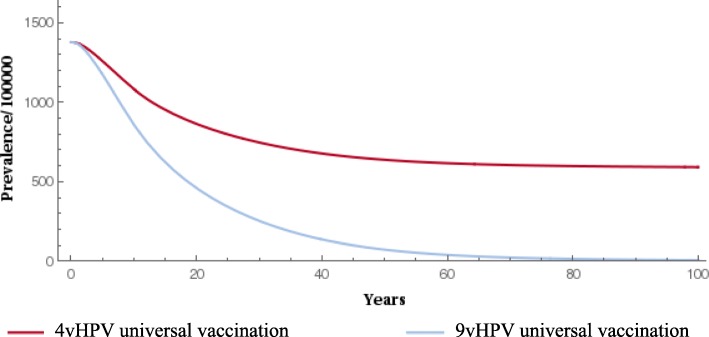
Epidemiological impact of two vaccination strategies on HPV16/18/31/33/45/52/58 infection prevalence among males over 100 years

**Fig. 3:**
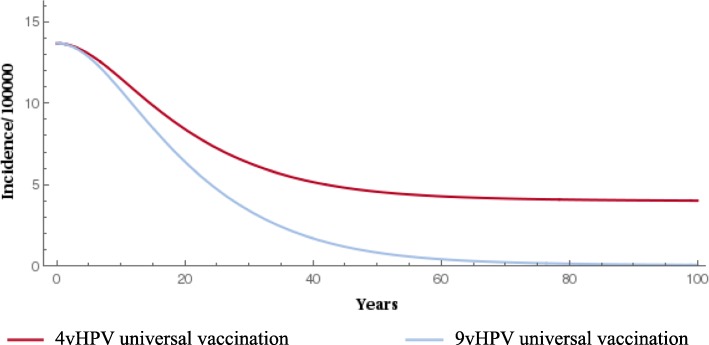
Epidemiological impact of two vaccination strategies on HPV16/18/31/33/45/52/58 related incidence of CIN1 over 100 years

**Fig. 4:**
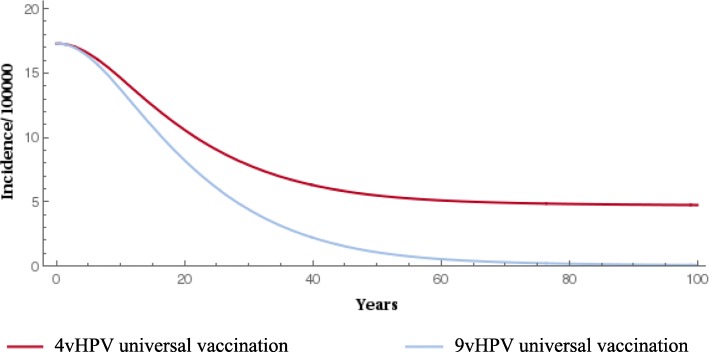
Epidemiological impact of two vaccination strategies on HPV16/18/31/33/45/52/58 related incidence of CIN2 over 100 years

**Fig. 5:**
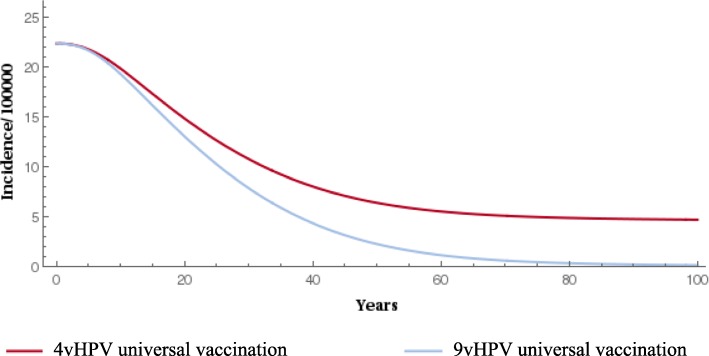
Epidemiological impact of two vaccination strategies on HPV16/18/31/33/45/52/58 related incidence of CIN3 over 100 years

**Fig. 6:**
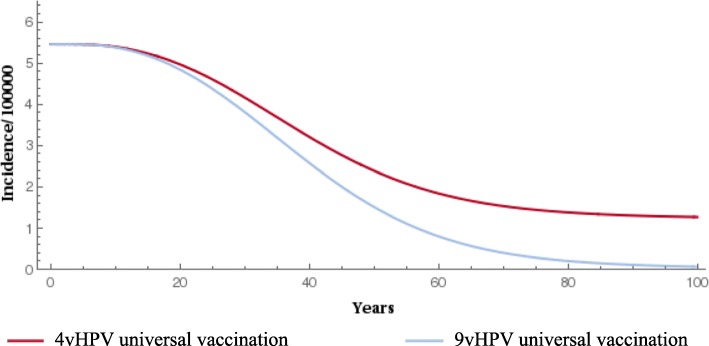
Epidemiological impact of two vaccination strategies on HPV16/18/31/33/45/52/58 related cervical cancer incidence over 100 years

**Fig. 7:**
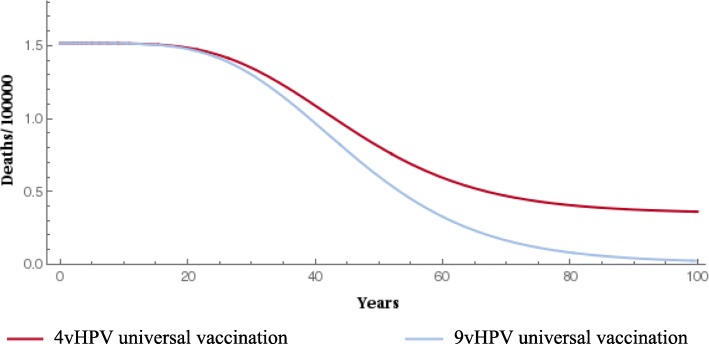
Epidemiological impact of two vaccination strategies on HPV16/18/31/33/45/52/58 related cervical cancer deaths over 100 years

When compared with the 4-valent vaccine, gender-neutral vaccination with the 9-valent vaccine significantly reduced the prevalence of HPV types 16/18/31/33/45/52/58 (Fig. 1 and Fig. 2). The 9-valent vaccine reduced the incidence of HPV type 16/18/31/33/45/52/58-related HPV infection among males and females and CIN1, CIN2, CIN3, cervical cancer cases and cervical cancer deaths, over 100 years, as shown in Table 2 and from Figs. 3, 4, 5, 6 and 7. The 9-valent vaccine would prevent additional 12,702 cases of CIN1, 15,000 CIN2 and 13,862 CIN3 over 100 years in comparison to the 4-valent vaccine, which corresponds respectively to a 50, 48 and 36% cumulative incidence decrease (Table 2). The 9-valent vaccine is also associated with an additional cumulative decrease of cervical cancer incidence of 24% compared with the 4-valent vaccine, corresponding to additional 2979 cervical cancer cases and 741 deaths avoided in females over 100 years (Table 2).

**Table 2:** Disease events and deaths prevented with the two vaccination strategies associated with cervical screening

HPV 16/18/31/33/45/52/58 related disease incidence cases and deaths	Years since start of vaccination programme		
	25	50	100
CIN1 cases- females			
4vHPV vs screening (nb of events avoided)	3125	11,579	31,382
9vHPV vs screening (nb of events avoided)	4278	16,146	44,084
9vHPV vs 4vHPV (nb of events avoided)	1153	4567	12,702
Cumulative incidence decrease, 9vHPV vs 4vHPV (%)	10.4	27.1	49.8
CIN2 cases- females			
4vHPV vs screening (nb of events avoided)	3948	14,852	40,551
9vHPV vs screening (nb of events avoided)	5321	20,254	55,552
9vHPV vs 4vHPV (nb of events avoided)	1373	5401	15,000
Cumulative incidence decrease, 9vHPV vs 4vHPV (%)	9.8	25.6	47.8
CIN3 cases- females			
4vHPV vs screening (nb of events avoided)	4232	18,227	54,031
9vHPV vs screening (nb of events avoided)	5224	22,789	67,893
9vHPV vs 4vHPV (nb of events avoided)	991	4562	13,862
Cumulative incidence decrease, 9vHPV vs 4vHPV (%)	5.2	16.1	35.5
Cervical cancer cases- females			
4vHPV vs screening (nb of events avoided)	242	2316	10,414
9vHPV vs screening (nb of events avoided)	305	2961	13,394
9vHPV vs 4vHPV (nb of events avoided)	63	644	2979
Cumulative incidence decrease, 9vHPV vs 4vHPV (%)	1.2	7.1	24.2
Cervical cancer deaths- females			
4vHPV vs screening (nb of deaths avoided)	16	399	2554
9vHPV vs screening (nb of deaths avoided)	20	512	3295
9vHPV vs 4vHPV (nb of events avoided)	4	113	741
Cumulative incidence decrease, 9vHPV vs 4vHPV (%)	0.3	4.1	19.7

The effect of 9-valent vaccination on HPV infection and precancerous cervical lesions incidence reduction is seen for an earlier time period than for cervical cancer. The reduction in incidence of HPV-related cancers and deaths from HPV-related cancers was more gradual, reflecting the fact that HPV-related cancers are diseases with slower progression (From Figs. 3, 4, 5, 6 and 7).

### Sensitivity analyses

**Fig. 8:**
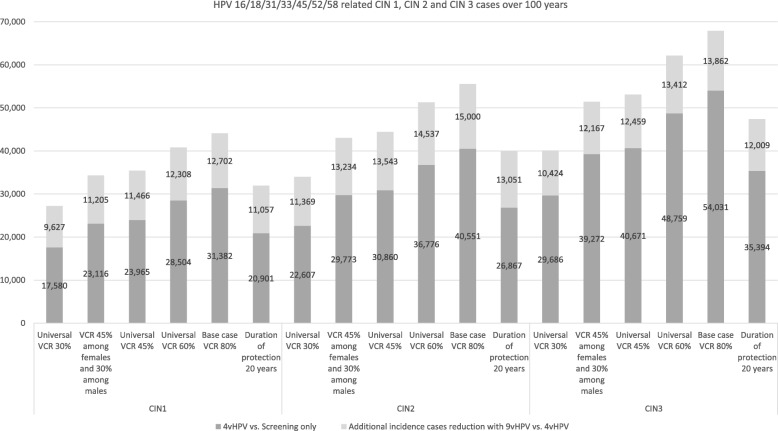
Sensitivity analyses results – CIN1 and CIN2/3 incidence cases prevented with the two vaccination strategies

**Fig. 9:**
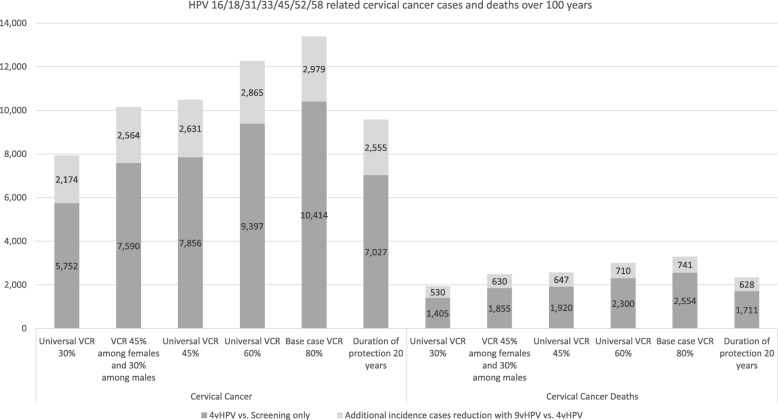
Sensitivity analyses results – Cervical cancer incidence cases and deaths prevented with the two vaccination strategies

The results of the sensitivity analyses, illustrated by the cumulative reduction in HPV 16/18/31/33/45/52/58 related disease incidence cases, are presented in 8 and 9. The base case (80% of maximum VCR) over 100 years following vaccination is associated with a cumulative reduction of 12,702 CIN1, 15,000 CIN2, 13,862 CIN3, 2979 cervical cancers and 741 cervical cancer deaths compared with a 4-valent vaccination strategy. When other scenarios were tested with lower vaccination coverage rates, ranging from 30 to 60%, a substantial cumulative reduction for all tested outcomes was still observed (Fig. 8 and Fig. 9). Furthermore, when reducing the duration of protection from lifetime to 20 years, the results showed that the number of additional cancer cases and deaths prevented with the 9-valent vaccination versus the 4-valent vaccination was still significant, with additional 628 cervical cancer deaths avoided and approximately 2555 cervical cancer cases, 11,057 CIN1 cases, 13,051 CIN2 and 12,009 CIN3 cases avoided compared with the 4-valent vaccination (Fig. 8 and Fig. 9). The 20 years duration protection is a very conservative approach in light of the real world data available for the 4-valent vaccines already today [44]. The results of this conservative analysis tend to show that the epidemiological outcomes are not very sensitive to the duration of protection since this parameter has a limited impact on the model results.

## Discussion

To our knowledge this is the first study assessing the epidemiological impact of a new vaccine strategy with the 9-valent vaccine in Switzerland. The analyses provide evidence for the epidemiological impact on cervical disease of a 9-valent vaccine in comparison to a 4-valent vaccine in a gender-neutral programme including girls and boys aged 11–26 years. The study was realized through collection and selection of the most relevant data to reflect the current clinical and epidemiological context in Switzerland, as closely as possible. The analyses show that 9-valent vaccination has a positive impact on the burden of HPV-related diseases in Switzerland. Assuming a maximum vaccination coverage of 80% in girls and boys aged 11 to 26 years, the 9-valent vaccination has the potential to additionally reduce, over 100-years following vaccination, cervical cancer incidence related to the 9 HPV types by 24%, cervical cancer deaths by 20%, CIN1 by 50%, CIN2 by 48% and CIN3 by 36%, respectively compared with a 4-valent vaccination strategy associated with cervical screening. Sensitivity analyses were conducted in order to test the impact of lower maximum vaccination coverage rates and a shorter duration of protection in the model outcomes. Results of these analyses are robust since a significant additional reduction in cervical cancer and precancerous lesions burden is still observed with the 9-valent vaccination compared with the 4-valent vaccination strategy.

Previous epidemiological models assessing gender-neutral HPV vaccination were already published in Europe. Ribassin-Majed et al. published in 2012 a deterministic transmission model comparing several vaccine scenarios in France. The authors observed that HPV vaccination may significantly decrease cervical cancer incidence with a stronger impact of gender-neutral vaccination considering the vaccine coverage in France in 2009 [45]. The epidemiological model from Choi et al. (2010) developed in the UK setting has shown that the 4-valent gender-neutral HPV vaccination was associated with an additional benefit in terms of reduction of cervical cancer and genital warts compared with a girls-only vaccination programme [46]. Other European epidemiological-focused published models in Germany [47], in Italy [48], in the Netherlands [49], and in Finland [50] have shown the positive epidemiological impact of gender-neutral HPV 2-valent and/or 4-valent vaccination. Besides, several cost-effectiveness studies recently published in Europe reported findings on the epidemiological impact of switching from a 4-valent vaccination to a 9-valent vaccination programme in a gender-neutral context [22–24]. For example, in Austria, a cost-effectiveness analysis conducted by Boiron et al. (2016) compared the epidemiological and economic outcomes of a 9-valent vaccination strategy versus a 4-valent vaccination programme in a gender-neutral context. This study showed that HPV-related diseases incidence could be reduced to a greater extent with the 9-valent vaccine (by 92% for HPV-related cervical cancers, by 96% for related CIN2/3 cases, by 83 and 76% for anal cancers respectively in females and males versus a screening-only strategy) compared with the 4-valent vaccine (incidence reduced by 75% for HPV-related cervical cancer, by 76% for related CIN2/3 cases, by 80 and 74% for anal cancers respectively in females and males versus a screening-only strategy). It represented 14,983 cases of CIN2/3 and 2544 cases of cervical cancer cases additionally prevented with the 9-valent vaccine over 100 years [22]. Similarly, a recent study examining the cost-effectiveness and epidemiological impact of gender-neutral vaccination with the 9-valent vaccine in Germany has also been published [23]. This analysis reported significant health benefit of a 9-valent vaccination compared with a 4-valent vaccination in terms of disease reduction: 24% reduction of cervical cancer incidence, 30 and 14% reduction of anal cancer incidence for males and females respectively, as well as over one million cases of genital warts avoided in 100 years [23]. Furthermore, an Italian study published by Mennini et al. in 2017 also assessed the cost-effectiveness of the 9-valent vaccine and reported the epidemiological impact of a switch from a 4-valent to a 9-valent vaccination [24]. The model results showed a significant diseases reduction in a gender-neutral programme with the 9-valent vaccine compared with the current vaccination programme with the 4-valent vaccine (reduction of 17% in the incidence of cervical cancer, 35 and 14% in anal cancer for males and females, and over a million cases of genital warts avoided after 100 years) [24].

While not the focus of this manuscript, the model used in this work also allowed us to estimate the cost-effectiveness of switching from the currently implemented HPV vaccination programme with the 4-valent vaccine to the 9-valent vaccine. Based on economic input parameters adapted to the Swiss setting [25] and considering the current private market price of the 4-valent vaccine, a price increase of 16 to 32% is estimated to be cost-effective based on ICER-thresholds used by NICE and WHO, respectively*.* The major strength is that the findings of this study are novel in the Swiss setting as previous epidemiological focused models and comparisons in specific European countries did not include the 9-valent versus the 4-valent vaccine among both males and females and were non-country-specific. Another strength of this analysis is the use of a dynamic model including herd immunity/protection effects.

The current analysis involves limitations. The first one is that some of the population-independent model inputs were not issued from Swiss-specific studies. However, to strengthen the study, all non-Swiss specific input values have been validated by expert opinion. A second limitation is that in order to simplify the calculations in the model, the epidemiological module simulated HPV types 6, 11, 16 and 18 separately, whereas the five additional types (31, 33, 45, 52 and 58) were combined into a single set of compartments. A third limitation was that this analysis only focused on the impact on cervical disease. While the main effect of a 9-valent HPV vaccination programme is predicted to occur for cervical disease, further incidence reduction is expected for other HPV-related diseases. The main difficulty encountered to adapt the model to the Swiss setting was to inform the VCR parameter. Indeed, a high heterogeneity of HPV vaccine coverage among cantons is observed in Switzerland. Uptake of HPV vaccination in 16 year old girls in Switzerland showed pronounced differences between different cantons ranging from 30 to 79% [21]. The maximum VCR used in the base case analysis was based on the assumption that the VCR in Switzerland would be higher than the observed rate in 2016 at the 9-valent vaccine launch and that it varied depending on gender and age of the vaccination (initial cohort or catch-up cohort) and the number of years after the 9-valent vaccine commercialization. To overcome the VCR variability between cantons and the results obtained in the base case analysis, sensitivity analyses on VCR parameter were conducted. The results showed that even with a low VCR of 30%, the 9-valent vaccine could greatly reduce the number of precancerous cervical lesions and cervical cancer cases and deaths and provided evidence that the 9-valent vaccine was still highly effective when compared with the 4-valent vaccine against HPV-related diseases. Riesen et al. published a dynamic transmission model to study the expected consequences of this spatial heterogeneity in vaccination uptake on the transmission and prevalence of HPV-16 in Switzerland. The authors concluded that spatial heterogeneity in HPV vaccination uptake is expected to diminish the effect of vaccination on HPV-16 prevalence, but the overall effect is small [51]. Thus, our findings are in line with the results by Riesen et al. [51].

It should be noted that several countries such as Ireland, Denmark and Japan experienced recently a sudden decline in VCR. For instance, in Ireland, the HPV vaccine uptake of the first dose dropped from 89.7% in 2014–2015 to 50% in 2016–2017 due to parental concerns about vaccine safety [52]. Although strong media campaigns are currently launched in Ireland to boost HPV vaccination, the former high levels of coverage rates are still not reached [52]. These recent events reveal the difficulty and time it takes to recover from a decline in VCR, reminding the necessity to support high HPV vaccination coverage rates in Swiss cantons.

A first model assessing the impact of HPV 4-valent vaccination has shown the effectiveness of HPV 4-valent vaccination in Switzerland by reducing HPV-related diseases burden in Switzerland [28]. An impact study was recently published and has demonstrated the short-term effectiveness of HPV vaccination with the 4-valent vaccine in a Swiss canton with a high VCR [53]. The data of our model suggest that replacing the current HPV 4-valent vaccination by the HPV 9-valent vaccination will further increase the impact on HPV-related diseases in Switzerland by reducing the burden of high-risk HPV genotypes other than HPV 16 or 18.

Real world data assessing the impact of HPV vaccination have demonstrated the public health impact on HPV infection, genital warts and cervical abnormalities. Garland et al. conducted a systematic review and have found reports for strong reductions in HPV types covered by the 4-valent HPV vaccine (up to 90%), for reductions of genital warts (up to 90%), for up to 45% reductions in low-grade cytological cervical abnormalities, and for up to 85% reductions in high-grade histologically proven cervical abnormalities. Even if the full public health potential of HPV vaccination is not yet realized, it can be easily expected that using 9-valent HPV universal vaccination will lead to an additional positive impact [44].

## Conclusion

This study showed how the switch from a gender-neutral vaccination programme with the 4-valent vaccine to the 9-valent vaccine impacts cervical disease in the Swiss setting. The switch to the 9-valent vaccination would yield significant incremental epidemiological benefits by further reducing cervical cancer and precancerous lesions in Switzerland.

## Data Availability

Written requests can be sent to the corresponding author. Model input data is publicly available [21, 22, 25–27, 29–38]. See methods section for details.

## Abbreviations

BAG: Bundesamt für Gesundheit/Federal Office of Public Health
BFS: Bundesamt für Statistik/Federal Office of Statistics
CIN: Cervical Intraepithelial Neoplasia
EKIF: Federal Vaccination Commission
HPV: Human papillomavirus
ICER: Incremental cost effectiveness ratio
NATSAL: National Survey of Sexual Attitudes and Lifestyles
NICER: National Institute for Cancer Epidemiology and Registration
RRP: Recurrent respiratory papillomatosis
US: United States
VCR: Vaccination coverage rate
